# Type and Severity of Migraine Determines Risk of Atrial Fibrillation in Women

**DOI:** 10.3389/fcvm.2022.910225

**Published:** 2022-05-31

**Authors:** Tae-Min Rhee, Eue-Keun Choi, Kyung-Do Han, Hyo-Jeong Ahn, So-Ryoung Lee, Seil Oh, Gregory Y. H. Lip

**Affiliations:** ^1^Department of Internal Medicine, Seoul National University Hospital, Seoul, South Korea; ^2^Department of Internal Medicine, Seoul National University College of Medicine, Seoul, South Korea; ^3^Department of Statistics and Actuarial Science, Soongsil University, Seoul, South Korea; ^4^Liverpool Centre for Cardiovascular Science, University of Liverpool and Liverpool Chest and Heart Hospital, Liverpool, United Kingdom; ^5^Department of Clinical Medicine, Aalborg University, Aalborg, Denmark

**Keywords:** migraine, atrial fibrillation, gender differences, women, migraine with aura

## Abstract

**Objective:**

To evaluate sex differences in the risk of atrial fibrillation (AF) according to the type and severity of migraine.

**Methods:**

We analyzed the nationwide health screening recipients in 2009 without previous AF diagnosis from the Korean National Health Insurance Service data. The diagnosis, type, and severity of migraine were determined using claims data. Newly developed AF was identified during a 10-year follow-up. Sex-difference in the effect of migraine on AF was evaluated.

**Results:**

A total of 4,020,488 subjects were enrolled from January 1, to December 31, 2009 and followed-up through December 31, 2018; 4,986 subjects had migraine with aura (age 50.6 ± 14.0 years, men 29.3%); and 105,029 had migraine without aura (age 51.6 ± 14.3 years, men 30.9%). Risk of AF in a mild degree of migraine was similar to that in the control group, regardless of sex or the presence of aura. Severe migraine without aura modestly but significantly increased the risk of AF in both men and women compared to controls, with increase in AF risk being most prominent in women who had severe migraine with aura [incidence rate (*IR*) = 3.39, hazard ratio (*HR*)_adjust_ = 1.48, 95% confidence intervals (*CI*) = 1.18–1.85]. No significant association according to aura was observed in men with severe migraines (*p* for interaction 0.011).

**Conclusion:**

Severe migraine with aura significantly increased the risk of incident AF in women, but not in men. Surveillance for incident AF and prompt lifestyle modification may be beneficial, particularly for young women suffering from severe migraine with aura.

## What is Already Known about the Issue?

Migraine, when accompanied with aura, is closely linked to ischemic stroke, particularly in women. Atrial fibrillation (AF) has been suggested as a possible explanation for the link between migraine and ischemic stroke. There is little evidence on the long-term risk of AF according to the type or severity of migraine or regarding sex differences.

## What this Study Adds to the Existing Database?

In this large-scale population-based study, women with severe migraine with aura had the highest chance of developing AF in the future (48% increase over the control group), while migraine with aura had no effect on the development of AF in men. A mild degree of migraine did not have any significant effect on the risk of AF development, regardless of sex, or the presence of accompanying aura. Severe migraine without aura raised the long-term risk of AF by 16–21% in both men and women.

## What are its Clinical Implications and What is Yet to be Explored in the Future?

We suggest that active surveillance for incident AF, combined with a lifestyle modification, could be advantageous, especially for young women who suffer from severe migraine with aura. Mechanisms that may explain sex differences in migraine’s adverse effect on AF should be further investigated, and the generalizability of our findings in other ethnic groups should be evaluated.

## Introduction

Migraine is a primary headache disease that can present with several neurological and autonomic symptoms, which are commonly prevalent in young to middle-aged women (1). When accompanied by aura, migraine has a profound effect on the quality of life due to the severity of symptoms; furthermore, it can be closely associated with ischemic stroke, especially in women (2). Various mechanisms have been suggested to explain the relationship between migraine and ischemic stroke, such as their common cardio-cerebrovascular risk factors, genetic predisposition, and the tendency to promote platelet aggregation, blood coagulation, and vascular intimal dysfunction in migraineurs (3).

The major cause of ischemic stroke is atrial fibrillation (AF). Because of intracardiac thromboembolism, AF has also been considered an important factor that can explain the association between migraine and ischemic stroke. The significant associations between AF and migraine with aura have been suggested in recent epidemiological studies in European and US populations (3, 4). However, there is little evidence on the long-term risk of AF according to the type or severity of migraine, or regarding sex differences. Furthermore, although the prevalence and disease burden of migraine in Asian populations differs significantly from those in Western countries (5).

Thus, we sought to evaluate the sex-specific risk of AF development according to the subgroups of migraine classified by type and severity, using data from the Korean nationwide health screening cohort, incorporating more than 4 million participants.

## Materials and Methods

### Data Source and Study Population

We utilized a nationwide health screening data from the Korean National Health Information Database (NHID) in the present study. The structure and characteristics of the data source were previously described elsewhere (6). To describe briefly, the NHID contains claims-based comprehensive data incorporating demographics, socioeconomic status, medical treatments and procedures, and disease diagnoses according to the 10th revised code of the International Classification of Diseases (ICD-10), including the entire Korean population of approximately 52 million in 2019. All insured subjects registered in the NHID are recommended to undergo biennial general health check-ups. The results from the national health screening, such as self-reporting questionnaires of previous medical history and lifestyle factors, anthropometric measurements, physical examinations, and laboratory data, were incorporated into the national health screening database as part of the NHID. Since all patients’ records and information were completely anonymized and de-identified before cohort establishment, informed consent could not be obtained, and this study protocol was exempted after review by the Seoul National University Hospital Institutional Review Board (E-2008-076-1147).

### Patient and Public Involvement

It was not possible to involve patients or the public in the design, or conduct, or reporting, or dissemination plans of our research.

### Establishment of Study Cohort

**FIGURE 1:**
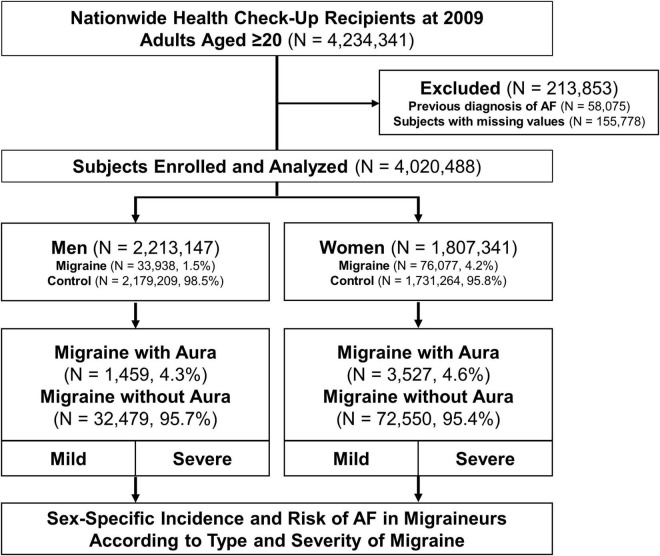
Study flow. Flow of establishment of the study population is shown. AF, atrial fibrillation.

From the national health screening data, a total of 4,234,341 participants aged ≥ 20 years who had undergone a nationwide general health check-up between 1st January and 31st December 2009, were screened initially (Figure 1). Participants with a previous history of AF before 31st December 2008 were excluded from the study. Among 4,020,488 subjects, patients with migraines were identified by more than one occasion of outpatient or inpatient diagnosis within 1 year, with dedicated ICD-10 codes registered by the physicians. The entire cohort was then divided into groups according to sex and the presence of migraine and was followed up for a median of 9.31 years (Q1–Q3, 9.12–9.58 years) until December 2018. The effect of migraine on the long-term risk of incident AF was evaluated according to the type (migraine with or without aura) and severity (mild or severe migraine) of migraine.

### Definitions of Comparison Groups, Study Outcomes, and Comorbidities

The detailed definitions of the comparison groups, covariates, and outcomes of interest are presented in Supplementary Table 1. We subdivided the patients having migraines into two groups according to the type and severity of migraines. Migraine was defined as the ICD-10 code of G43, in which migraine with aura was defined as G43.1 and migraine without aura as the rest (7). Severe migraine was defined as following: (1) Migraine requiring medical attention (e.g., admission or visit to the emergency department due to migraine) or active therapeutic intervention (e.g., migraine-specific medications, neuromodulations, or nerve blocks) and (2) chronic migraine that persists for more than 3 months. The outcome of interest was the occurrence of incident AF during the follow-up period. Comorbidities such as diabetes mellitus (DM), hypertension, and dyslipidemia, were defined by ICD-10 codes as described and validated in previous studies by our group (6, 8, 9).

### Statistical Analysis

Data are presented as numbers and relative frequencies (percentages) for categorical variables and as mean ± standard deviation for continuous variables. For comparison, the chi-square test and the independent sample *t*-test or analysis of variance test were used according to the requirement. Incidence rates (*IRs*) of AF were described as the number of events per 1,000 person-years. Cumulative event rates were compared among groups according to the type and/or severity of migraine using Kaplan–Meier censoring estimates and the log-rank test. Hazard ratios (*HRs*) with 95% confidence intervals (*CI*), were calculated using univariate and multivariate Cox proportional hazard models. The adjusted *HRs* were calculated using a multivariate model that included the following covariates: age, smoking habit, level of alcohol consumption, regular physical activity, low household income, body mass index (BMI), DM, hypertension, dyslipidemia, and estimated glomerular filtration rate. Subgroup analyses divided by age, BMI, smoking habit, DM, hypertension, and dyslipidemia were subsequently performed. All analyses were performed separately for men and women. All *p*-values were two-sided, and a value of < 0.05 was considered statistically significant. Statistical analyses were performed using SAS version 9.4 (SAS Institute, Cary, NC, United States) and Stata statistical software release 14 (StataCorp, College Station, TX, United States).

## Results

Baseline characteristics according to the type of migraine are presented in the total study population (Supplementary Table 2) and stratified by sex (Table 1); a total of the 4,020,488 participants (men, *n* = 2,213,147; women, *n* = 1,807,341), the prevalence of migraine was 1.5% (*n* = 33,938) in men and 4.2% (*n* = 76,077) in women. The proportion of migraine with aura, in the migraine group, was similar between men and women (men, 4.3%; women, 4.6%). The average age of the migraine group was higher than that of the control group, and patients who had migraines without aura were slightly older than those who presented with aura. According to the presence of migraine and the type of migraine, lifestyle factors and comorbidities were similar among men and women. The migraine group showed a more favorable lifestyle than the control group regarding the status of smoking and drinking habits; however, there was no significant difference seen in the status of physical activity between the two groups. In the migraine group, regardless of the presence of aura, the presence of other comorbidities such as DM, hypertension, dyslipidemia, and depressive disorder was higher than in the control group.

**TABLE 1:** Baseline characteristics of the study population.

	Men				Women			
	Migraine with aura (*N* = 1,459)	Migraine without aura (*N* = 32,479)	Control (*N* = 2,179,209)	*P*	Migraine with aura (*N* = 3,527)	Migraine without aura (*N* = 72,550)	Control (*N* = 1,731,264)	*P*
Age, years	49.5 ± 14.7	50.1 ± 14.8	45.5 ± 13.5	< 0.001	51.1 ± 13.7	52.3 ± 14.0	48.5 ± 14.5	< 0.001
Body mass index, kg/m^2^	24.3 ± 3.1	24.2 ± 3.0	24.1 ± 3.1	0.004	23.5 ± 3.3	23.5 ± 3.3	23.2 ± 3.3	< 0.001
Smoking status, n (%)				< 0.001				< 0.001
Non-smoker	521 (35.7)	11,394 (35.1)	661,814 (30.4)	–	3,351 (95.0)	69,058 (95.2)	1,638,428 (94.6)	–
Ex-smoker	418 (28.7)	9,038 (27.8)	532,724 (24.5)	–	66 (1.9)	1,151 (1.6)	32,805 (1.9)	–
Current smoker	520 (35.6)	12,047 (37.1)	984,671 (45.2)	–	110 (3.1)	2,341 (3.2)	60,031 (3.5)	–
Drinking habit, n (%)				< 0.001				< 0.001
Non-drinker	623 (42.7)	13,658 (42.1)	698,406 (32.1)	–	2,798 (79.3)	58,370 (80.5)	1,290,002 (74.5)	–
Mild drinker	679 (46.5)	15,026 (46.3)	1,183,476 (54.3)	–	691 (19.6)	13,556 (18.7)	422,015 (24.4)	–
Heavy drinker	157 (10.8)	3,795 (11.7)	297,327 (13.6)	–	38 (1.1)	624 (0.9)	19,247 (1.1)	–
Regular physical activity, n (%)	316 (21.7)	6,678 (20.6)	439,120 (20.2)	0.067	561 (15.9)	11,269 (15.5)	270,170 (15.6)	0.769
Low income status, n (%)	294 (20.2)	5,610 (17.3)	386,613 (17.7)	0.005	928 (26.3)	18,617 (25.7)	448,431 (25.9)	0.297
eGFR, mL/min/1.73m^2^	88.8 ± 28.6	87.9 ± 28.1	92.9 ± 28.8	< 0.001	78.3 ± 24.3	77.4 ± 25.4	80.5 ± 26.0	< 0.001
***Comorbidities and risk factors***								
Diabetes mellitus, n (%)	171 (11.7)	3,724 (11.5)	211,206 (9.7)	< 0.001	265 (7.5)	6,323 (8.7)	125,950 (7.3)	< 0.001
Hypertension, n (%)	550 (37.7)	12,063 (37.1)	587,607 (27.0)	< 0.001	1,170 (33.2)	25,861 (35.7)	441,536 (25.5)	< 0.001
Dyslipidemia, n (%)	329 (22.6)	6,902 (21.3)	357,027 (16.4)	< 0.001	884 (25.1)	19,107 (26.3)	339,658 (19.6)	< 0.001
Depressive disorder, n (%)	11 (0.8)	288 (0.9)	3,762 (0.2)	< 0.001	82 (2.3)	1,024 (1.4)	7,833 (0.5)	< 0.001

*GFR, estimated glomerular filtration rate.*

### Incidence and Risk of Atrial Fibrillation in Migraineurs

Supplementary Table 3 presents the number of events during the follow-up period, calculated *IRs*, and adjusted and unadjusted *HRs* for AF according to the type and severity of migraine. The presence of migraine (*HR*_adjust_ = 1.10, 95% *CI* = 1.06–1.14), especially in migraine without aura [*HR*_adjust_ = 1.10 (1.06–1.14)], slightly elevated the risk of AF after adjustment. Severe migraine significantly increased the risk of AF in migraineurs without aura [*HR*_adjust_ = 1.13 (1.08–1.18)] but was not statistically significant in those with aura [*HR*_adjust_ = 1.13 (0.93–1.39)]. A mild degree of migraine, irrespective of the type of migraine, was not associated with an increased risk of AF after adjustment (Supplementary Figure 1).

### Sex-Difference in the Risk of Atrial Fibrillation According to the Type and Severity of Migraine

**FIGURE 2:**
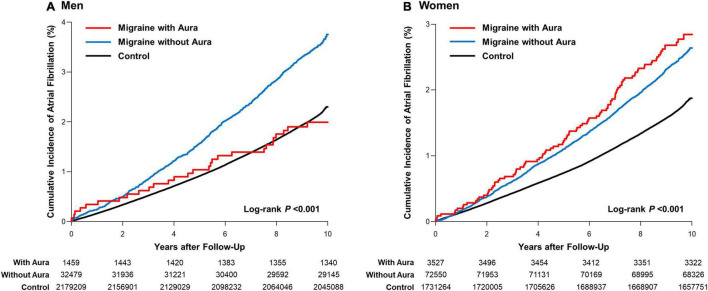
Sex-stratified cumulative incidence of atrial fibrillation in migraine patients. Cumulative hazard curves for AF according to the type of migraine in **(A)** men and **(B)** women.

According to the type and severity of migraine, sex-stratified results of AF risk are presented in Figure 2, Supplementary Figure 2, and Table 2. In both men and women with mild migraine, irrespective of the type of migraine, the adjusted risk of AF was similar to that in the control group (Supplementary Figure 3). In men, the *IRs* and adjusted *HRs* of AF modestly increased in the group, including patients who had migraine without aura [*IR* = 3.72/1,000 person-years vs. 2.20 in the control group, *HR*_adjust_ = 1.15 (1.08–1.22)], which further increased when combined with severe migraine [*IR* = 4.51/1,000 person-years, *HR*_adjust_ = 1.21 (1.12–1.31)]. A similar increase in the incidence and risk of AF was observed in women migraineurs without aura [*IR* = 2.63/1,000 person-years vs. 1.80 in the control group, *HR*_adjust_ = 1.13 (1.08–1.19); for severe degree, *IR* = 3.00, *HR*_adjust_ = 1.16 (1.09–1.22)].

**TABLE 2:** Sex-stratified risk of atrial fibrillation according to type and severity of migraine.

				Men				Women		
			Event/N	IR, per 1,000 p-y	Unadjusted HR (95% CI)	*Adjusted HR (95% CI)	Event/N	IR, per 1,000 p-y	Unadjusted HR (95% CI)	*Adjusted HR (95% CI)
Control group			43,598/2,179,209	2.20	1 (Reference)	1 (Reference)	28,664/1,731,264	1.80	1 (Reference)	1 (Reference)
Migraine group			1,122/33,938	3.65	1.64 (1.55–1.74)	1.13 (1.06–1.20)	1,869/76,077	2.64	1.45 (1.39–1.52)	1.14 (1.09–1.20)
	Migraine without aura		1,094/32,479	3.72	1.68 (1.58–1.78)	1.15 (1.08–1.22)	1,773/72,550	2.63	1.44 (1.38–1.52)	1.13 (1.08–1.19)
		Mild degree	413/15,648	2.89	1.30 (1.18–1.43)	1.07 (0.97–1.18)	473/25,819	1.96	1.08 (0.99–1.18)	1.07 (0.97–1.17)
		Severe degree	681/16,831	4.51	2.03 (1.88–2.19)	1.21 (1.12–1.31)	1,300/46,731	3.00	1.65 (1.56–1.74)	1.16 (1.09–1.22)
	Migraine with aura		28/1,459	2.09	0.94 (0.65–1.36)	0.65 (0.45–0.95)	96/3,527	2.93	1.61 (1.31–1.96)	1.41 (1.15–1.72)
		Mild degree	11/641	1.86	0.84 (0.47–1.52)	0.70 (0.39–1.26)	19/1,085	1.88	1.04 (0.66–1.62)	1.18 (0.75–1.84)
		Severe degree	17/818	2.28	1.02 (0.63–1.64)	0.63 (0.39–1.01)	77/2,442	3.39	1.86 (1.49–2.32)	1.48 (1.18–1.85)

*The adjusted HRs were calculated by multivariate model including covariates as follows: age, smoking habit, alcohol consumption level, regular physical activity, low household income level, body mass index, diabetes mellitus, hypertension, dyslipidemia, and estimated glomerular filtration rate. CI, confidence interval; HR, hazard ratio; IR, incidence rate; p-y, person-years.

**FIGURE 3:**
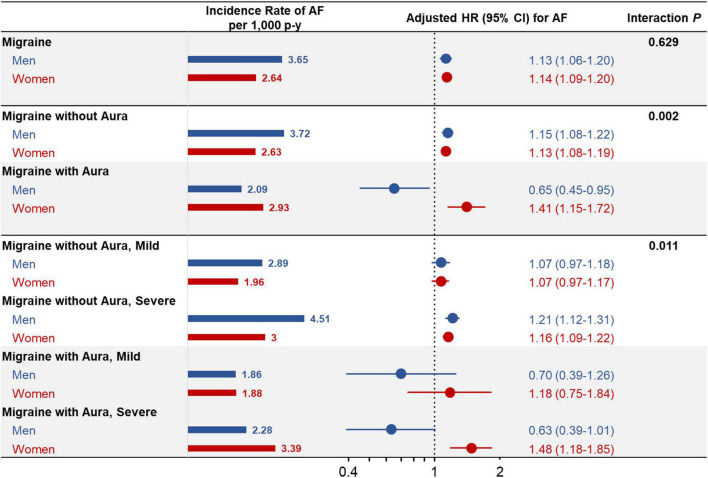
Sex-stratified comparison of risk of atrial fibrillation according to type and severity of migraine. The incidence rate of and multivariate adjusted *HRs* with 95% *CIs* for AF according to the type and severity of migraine are presented stratified by men and women. CI, confidence interval; HR, hazard ratio; p-y, person-year; otherwise, as shown in Figure 1.

In contrast, the presence of a severe degree of migraine with aura did not significantly affect the incidence and risk of AF in men. In women, the AF incidence and risk were markedly increased in those who had migraine with aura [*IR* = 2.93/1,000 person-years, *HR*_adjust_ = 1.41 (1.15–1.72)], with an additional increase in the risk of AF in women having severe degrees of migraine [*IR* = 3.39/1,000 person-years, *HR*_adjust_ = 1.48 (1.18–1.85)]. Significant interactions were observed between men and women for the HRs for AF, according to the type and severity of migraine (Figure 3).

### Subgroup Analysis

A sex-stratified analysis of various exploratory subgroups is shown in Supplementary Table 4. The adjusted effect of severe migraine with aura on the risk of AF was particularly prominent in young to middle-aged women aged < 65 years [*HR*_adjust_ = 1.69 (1.22–2.36)] and normal-weight women with BMI < 25 kg/m^2^ [*HR*_adjust_ = 1.61 (1.21–2.15)].

In both men and women, the presence of DM, dyslipidemia, and smoking status did not significantly alter the results of the total study population. The adjusted *HRs* of AF in patients having severe migraines with aura, compared to the control group, ranged from 1.4 to 1.5 in low-risk women without the traditional risk factors.

## Discussion

To the best of our knowledge, this is the first study to provide sex-stratified results regarding the effect of the type and the severity of migraine on AF development. In this nationwide cohort of 4 million health screening examinees, we found that (1) mild migraine did not have any significant effect on the risk of AF regardless of sex or the presence of accompanying aura; (2) severe migraine without aura increased the long-term risk of AF by 16–21% in both men and women; and (3) women who had severe migraine with aura were at the highest risk for AF in the future, while the effect of migraine with aura on the development of AF was not significant in men.

### Role of Atrial Fibrillation on the Risk of Ischemic Stroke in Migraineurs

Migraine is a common neurological disorder with a lifetime prevalence ranging from 10 to 20% and is well known for its close association with ischemic stroke, especially when accompanied by an aura (2). A recent result from the Atherosclerosis Risk in Communities (ARIC) study, which analyzed the risk of stroke by subtype in 1,622 migraineurs with a mean age of 60 years, reported a high cardioembolic stroke risk [*HR* = 3.7 (1.6–8.7)] in migraine with visual aura (10). It is also known that the prevalence of patent foramen ovale (PFO) (11) and the risk of venous thromboembolism (7) are high in subjects having migraines with aura. However, in young European adults, migraine with aura was significantly associated with cryptogenic ischemic stroke, regardless of the presence of PFO (12).

In this regard, epidemiological evidence has recently been presented on the role of AF in the association between migraine and ischemic stroke. A Danish nationwide population-based study was the first to suggest a positive association between migraine and the occurrence of AF (3). The ARIC study group also reported the possibility that an increase in stroke risk caused by migraine with aura would be mediated by AF (4). In the present study, in contrast to the previous studies, the association between migraine and AF was modest in the total population combining men and women. We found that a mild degree of migraine did not affect the future risk of AF, regardless of the presence of aura. Interestingly, in the case of severe migraines requiring medical attention, migraines without aura moderately increased the risk of AF in both men and women. This result is in line with a previous study that suggested that patients with severe migraines who required inpatient treatment or who visited the emergency department had a higher risk of cardiovascular events than those with mild migraines (3).

Several mechanisms have been suggested to explain the increase in the risk of AF in patients with severe migraines. Severely symptomatic migraine with aura is known to share genetic predispositions with stroke (13). Although the common genetic risk or causal relationship between the type or severity of migraine and AF has not yet been completely elucidated (14), one study has reported that a certain subtype of stroke that is strongly correlated with AF, e.g., multifocal embolic infarction, is closely related to migraine with aura (15). Furthermore, migraineurs have a high blood concentration of calcitonin gene-related peptide, a major neuropeptide involved in an acute migraine attack, which is known to induce blood flow alteration through coronary vasodilation (16). Accordingly, it has been hypothesized that repetitive migraine attacks may contribute to the occurrence of AF (15). Sharing common inflammatory factors (17) or the electrophysiological mechanism, including cortical spreading depolarization, can also affect the occurrence of AF (18). There have also been reports, which state that the repeated use of non-steroidal anti-inflammatory drugs for severe migraine may contribute to an increased risk of AF (19). Further studies are crucial to reveal causality and mechanisms of the development of AF in migraineurs.

### Sex Difference in the Long-Term Risk of Atrial Fibrillation Affected by Type and Severity of Migraine

Migraines have sex-dependent epidemiology that shows a rapid increase of incidence in women after puberty (20). The previous research on migraine and various cardiovascular diseases (CVD) among women has been continuously performed, e.g., the Women’s Health Study or the Nurses’ Health Study (21). Epidemiologic evidence is robust for the high risk of ischemic stroke, particularly in young to middle-aged women having migraine with aura (22). On the other hand, reports on the risk of CVD according to migraine and its subtypes in men are limited and inconsistent (23).

Male migraineurs are considered to have a high risk of myocardial infarction, while the risk of stroke is relatively low (24). In a Taiwanese nationwide cohort study, the increase in venous thromboembolism risk caused by migraine with aura was pronounced in women [*HR*_adjust_ = 2.81 (1.41–5.58)], but not in men [*HR*_adjust_ = 1.81 (0.72–4.550)] (7). In contrast, as a result of a 20-year follow-up in the ARIC study, the migraine with aura group showed a significant increase in AF incidence in men, but no effect was seen in women (4). Therefore, it is essential to focus on the sex differences in migraineurs and provide epidemiological data on cardiovascular outcomes, especially for men.

To the best of our knowledge, the present study is the first to report significant gender differences in AF risk, especially in the group having severe migraines with aura. The relatively low risk of AF in male patients having migraines with aura should be interpreted with caution, since the incident rate was nearly identical to that of the control group, with insignificant unadjusted *HR*. Early medical attention in male migraineurs with subsequent lifestyle modifications, such as abstinence from alcohol or smoking cessation, may have affected this result. Since there is a lack of previous pre-clinical or clinical evidence that supports the decreased risk of AF in male migraineurs; our results could be hypothesis-generating at best, which requires further validation in future studies.

To date, no clear mechanisms have been established regarding sex differences related to the adverse cardiovascular effects of migraine (25). Differences in genetic predisposition and the distribution of risk factors between men and women according to ethnicity may have been involved. The well-known sex differences in the distribution of comorbid conditions in migraineurs may also be an important factor, e.g., anxiety, depression, fibromyalgia, or endometriosis, which are predominant in women, whereas obesity, coronary atherosclerosis, or diabetes are predominant in men (26). In this regard, estrogen levels, which are known to be generally low in migraineurs, may reduce the protective effect for CVD and increase AF risk in women. This interaction of fluctuating levels of sex hormones may also explain the prominent sex differences in the risk of migraine-related AF (25), which remains to be investigated in future studies.

### Clinical Implications

The significant AF risk observed in women having severe migraines with aura, despite their young age, should not be neglected, considering the long-term disease burden associated with subsequent stroke risk. Indeed, it may be important to avoid oral contraceptive use and maintain a healthy lifestyle such as smoking cessation (27). All this is aligned with the current approach to the holistic or integrated management of AF, which is associated with improved outcomes (28, 29).

Thus, a regular cardiovascular assessment, including electrocardiogram monitoring, is warranted, and a prompt referral to the cardiologist will be helpful if necessary. The application of wearable devices has been recently suggested as a new paradigm for AF surveillance with improved efficacy and accessibility, which may also be considered for this population (30).

Meanwhile, most of the previous evidence has relatively overlooked the clinical significance of migraines without aura (7, 10). In the present study, a modest but significant increase in the future risk of AF was observed in severe migraineurs even without aura. Evaluation of the associated risk factors and surveillance for AF may also be considered in patients with significant migraine without aura, requiring medical attention. In particular, considering that the highest crude incidence rate of AF in men was observed in those with a severe degree of migraine without aura, physicians should not underestimate the risk of future AF in the men who are suffering from migraine, even without aura.

### Study Limitations

The present study had several limitations. First, due to the innate limitation of this retrospective observational cohort study, the causality of the sex-specific association between migraine and AF found in this study could not be verified. Second, although the adjusted results for the possible confounders were presented by multivariate regression analysis, the influence of residual or unmeasured confounding factors cannot be ignored. Third, the working definition utilizing the ICD-10 diagnostic code was used to define covariates and outcomes in the present study, which raises the possibility of misclassification bias or bias due to under- or over-reporting. Especially, we could not acquire the information on the specific type of AF (paroxysmal, persistent, or permanent) or the possibility of accompanied organic heart disease. Finally, since the present study was conducted only on the Korean population, one of the most homogeneous ethnic populations in the world, it is necessary to further evaluate the generalizability of our results in various Asian populations and other ethnicities in future studies.

## Conclusion

Severe migraine without aura mildly increased the risk of AF without sex difference, while severe migraine with aura significantly increased the risk of AF in women, but not in men. Active surveillance for the incident AF with a recommendation for lifestyle modification may be beneficial, particularly for young women who have severe migraines with aura.

## Data Availability Statement

The datasets presented in this article are not readily available because the dataset is administrated by the Korean National Health Insurance service and is only accessible by the authorized investigators. Requests to access the datasets should be directed to E-KC, choiek17@snu.ac.kr.

## Ethics Statement

The studies involving human participants were reviewed and approved by the Seoul National University Hospital Institutional Review Board (E-2008-076-1147). Written informed consent for participation was not required for this study in accordance with the national legislation and the institutional requirements.

## Author Contributions

T-MR and E-KC: conception and design and writing the manuscript. T-MR, K-DH, H-JA, and E-KC: acquisition of data. T-MR, K-DH, S-RL, and E-KC: analysis and interpretation of data. H-JA, S-RL, SO, and GL: administrative, technical, and material supports. E-KC: study supervision. All authors contributed to the article and approved the submitted version.

## Conflict of Interest

E-KC Research grants or speaking fees from Bayer, BMS/Pfizer, Biosense Webster, Chong Kun Dang, Daiichi-Sankyo, Dreamtech Co., Ltd., Medtronic, Samjinpharm, Sanofi-Aventis, Seers Technology, and Skylabs. GL Consultant and speaker for BMS/Pfizer, Boehringer Ingelheim, and Daiichi-Sankyo. No fees were received personally. No other relationships or activities that could appear to have influenced the submitted work. The remaining authors declare that the research was conducted in the absence of any commercial or financial relationships that could be construed as a potential conflict of interest.

## Publisher’s Note

All claims expressed in this article are solely those of the authors and do not necessarily represent those of their affiliated organizations, or those of the publisher, the editors and the reviewers. Any product that may be evaluated in this article, or claim that may be made by its manufacturer, is not guaranteed or endorsed by the publisher.

## Abbreviations

AF: atrial fibrillation
ARIC: Atherosclerosis Risk in Communities
BMI: body mass index
CI: confidence intervals
CVD: cardiovascular diseases
DM: diabetes mellitus
ICD-10: 10th revised code of the International Classification of Diseases
HR: hazard ratio
IR: incidence rate
NHID: National Health Information Database
PFO: patent foramen ovale.
