# Supplementary material for: Type and Severity of Migraine Determines Risk of Atrial Fibrillation in Women
Source: Front Cardiovasc Med. 2022 May 31;9:910225. doi: 10.3389/fcvm.2022.910225 (PMC9197451; doi:10.3389/fcvm.2022.910225)
Supplement: Supplementary file 1 [file Data_Sheet_1.pdf]

Supplemental Materials

Type and Severity of Migraine Determines Long-Term Risk of Atrial Fibrillation in Women: A Nationwide Population-Based Study

Contents of the Supplementary Appendix

I. Supplementary Tables

II. Supplementary Figures

I. Supplementary Tables

Table S1. List of the definitions of covariates and outcomes

	ICD-10-CM code and definition	Diagnostic definition
**Inclusion/exclusion criteria**		
**Atrial fibrillation**	I48.0-48.4, I48.9	Admission≥1 or outpatient department≥2
**Migraine**	G43 (migraine with aura, G43.1)	Admission≥1 or outpatient department≥2
**Comorbidities**		
**Diabetes mellitus**	E11-E14; and minimum 1 prescription of anti-diabetic drugs (sulfonylureas, metformin, meglitinides, thiazolidinediones, dipeptidyl peptidase-4 inhibitors, α-glucosidase inhibitors, and insulin).	Admission ≥1 or outpatient department ≥2
	or fasting glucose level ≥ 126 mg/dL	Based on the results of health exam
**Hypertension**	I10-I13, I15; and minimum 1 prescription of anti-hypertensive drug (thiazide, loop diuretics, aldosterone antagonist, alpha-/beta-blocker, calcium-channel blocker, angiotensin-converting enzyme inhibitor, angiotensin II receptor blocker).	Admission ≥1 or outpatient department ≥2
	or systolic/diastolic blood pressure ≥ 140/90 mmHg	Based on the results of health exam
**Dyslipidemia**	E78	Admission or outpatient department ≥1
	or Total cholesterol ≥ 240 mg/dL	Based on the results of health exam
**Depressive disorder**	F33	Any of hospitalization or outpatient visit ≥1
**Health exam questionnaires**		
**Heavy alcoholics (estimated amount of alcohol intake per week)**	Non (0 g) / Mild (0< g <210) / **Heavy (≥210 g)**	Based on the results of health exam
**Current smokers**	Smoking habits : Nonsmoker / Ex-smoker / **Current smoker**	Based on the results of health exam
**Regular physical activity**	Performing a moderate physical activity more than 30 minutes at least 5 times per week or strenuous physical activity more than 20 minutes at least 3 times per week	Based on the results of health exam
**Low income status**	Income lowest 20% and medical aid	Based on the results of health exam
**Outcome**		
**Atrial fibrillation**	I48.0-48.4, I48.9	Admission≥1 or outpatient department≥2

Table S2. Baseline characteristics of the total study population

	Migraine with aura (N = 4,986)	Migraine without aura (N = 105,029)	Control (N = 3,910,473)	*P*
Age, years	50.6 ± 14.0	51.6 ± 14.3	46.8 ± 14.0	<.001
Men, n (%)	1,459 (29.3)	32,479 (30.9)	2,179,209 (55.7)	<.001
Body mass index, kg/m^2	23.7 ± 3.3	23.7 ± 3.2	23.7 ± 3.2	.014
Smoking status, n (%)				<.001
Non-smoker	3,872 (77.7)	80,452 (76.6)	2,300,242 (58.8)	-
Ex-smoker	484 (9.7)	10,189 (9.7)	565,529 (14.5)	-
Current smoker	630 (12.6)	14,388 (13.7)	1,044,702 (26.7)	-
Drinking habit, n (%)				<.001
Non-drinker	3,421 (68.6)	72,028 (68.6)	1,988,408 (50.9)	-
Mild drinker	1,370 (27.5)	28,582 (27.2)	1,605,491 (41.1)	-
Heavy drinker	195 (3.9)	4,419 (4.2)	316,574 (8.1)	-
Regular physical activity, n (%)	877 (17.6)	17,947 (17.1)	709,290 (18.1)	<.001
Low income status, n (%)	1,222 (24.5)	24,227 (23.1)	835,044 (21.4)	<.001
eGFR, mL/min/1.73m^2	79.3 ± 9.2	79.6 ± 9.8	80.2 ± 9.5	<.001
Comorbidities and risk factors				
Diabetes mellitus, n (%)	436 (8.7)	10,047 (9.6)	337,156 (8.6)	<.001
Hypertension, n (%)	1,720 (34.5)	37,924 (36.1)	1,029,143 (26.3)	<.001
Dyslipidemia, n (%)	1,213 (24.3)	26,009 (24.8)	696,685 (17.8)	<.001
Depressive disorder, n (%)	93 (1.9)	1,312 (1.2)	11,595 (0.3)	<.001

Abbreviations: eGFR, estimated glomerular filtration rate.

Table S3. Risk of atrial fibrillation according to type and severity of migraine in total population

	Event / N	IR, per 1,000 p-y	Unadjusted HR (95% CI)	*Adjusted HR (95% CI)
Control group	72,262 / 3,910,473	2.02	1 (reference)	1 (reference)
Migraine group	2,991 / 110,015	2.95	1.44 (1.39-1.50)	1.10 (1.06-1.14)
Migraine without aura	2,867 / 105,029	2.96	1.45 (1.40-1.50)	1.10 (1.06-1.14)
Mild degree	886 / 41,467	2.31	1.13 (1.06-1.21)	1.05 (0.98-1.12)
Severe degree	1,981 / 63,562	3.39	1.66 (1.58-1.73)	1.13 (1.08-1.18)
Migraine with aura	124 / 4,986	2.68	1.31 (1.10-1.56)	1.08 (0.90-1.28)
Mild degree	30 / 1,726	1.87	0.92 (0.64-1.31)	0.93 (0.65-1.33)
Severe degree	94 / 3,260	3.11	1.52 (1.24-1.86)	1.13 (0.93-1.39)

*The adjusted HRs were calculated by multivariate model including covariates as follows: age, sex, smoking habit, alcohol consumption level, regular physical activity, low household income level, body mass index, diabetes mellitus, hypertension, dyslipidemia, and estimated glomerular filtration rate.

Abbreviations: CI, confidence interval; HR, hazard ratio; IR, incidence rate; p-y, person-years.

Table S4. Sex-stratified subgroup analysis

		Men				Women			
		Event/N	IR, per 1,000 p-y	Adjusted* HR (95% CI)	$P_{interaction}$	Event / N	IR, per 1,000 p-y	Adjusted HR (95% CI)	$P_{interaction}$
Age ≥65	Control	17,924/230,431	9.56	1 (reference)	.009	15,943/260,491	7.02	1 (reference)	<.001
	Aura (-), Mild	183/2,334	9.73	0.98 (0.85-1.13)		226/3,859	6.67	0.95 (0.83-1.08)	
	Aura (-), Severe	377/4,216	11.27	1.13 (1.02-1.25)		755/11,576	7.43	1.04 (0.97-1.12)	
	Aura (+), Mild	7/95	8.94	0.90 (0.43-1.90)		8/125	7.52	1.06 (0.53-2.13)	
	Aura (+), Severe	6/180	3.91	0.40 (0.18-0.88)		42/508	9.56	1.33 (0.98-1.80)	
Age <65	Control	25,674/1,948,778	1.43	1 (reference)		12,721/1,470,773	0.93	1 (reference)	
	Aura (-), Mild	230/13,314	1.85	1.15 (1.01-1.31)		247/21,960	1.19	1.20 (1.06-1.37)	
	Aura (-), Severe	304/12,615	2.59	1.34 (1.20-1.50)		545/35,155	1.64	1.34 (1.23-1.46)	
	Aura (+), Mild	4/546	0.78	0.52 (0.19-1.38)		11/960	1.22	1.29 (0.72-2.33)	
	Aura (+), Severe	11/638	1.85	0.92 (0.51-1.66)		35/1,934	1.91	1.69 (1.22-2.36)	
BMI ≥25	Control	17,419/808,467	2.36	1 (reference)	.061	12,386/465,664	2.9	1 (reference)	.167
	Aura (-), Mild	150/6,018	2.70	0.96 (0.82-1.12)		198/7,219	2.96	1.04 (0.90-1.19)	
	Aura (-), Severe	266/6,340	4.61	1.25 (1.11-1.41)		544/15,110	3.89	1.10 (1.01-1.20)	
	Aura (+), Mild	8/278	3.13	1.16 (0.58-2.32)		5/299	1.79	0.66 (0.27-1.57)	
	Aura (+), Severe	10/326	3.35	0.90 (0.48-1.67)		30/742	4.39	1.31 (0.91-1.87)	
BMI <25	Control	26,179/1,370,742	2.11	1 (reference)		16,278/1,265,600	1.4	1 (reference)	
	Aura (-), Mild	263/9,630	3.01	1.14 (1.01-1.29)		275/18,600	1.58	1.09 (0.96-1.22)	

	Aura (-), Severe	415/10,491	4.45	1.18 (1.07-1.30)		756/31,621	2.57	1.20 (1.11-1.29)	
	Aura (+), Mild	3/363	0.90	0.33 (0.11-1.04)		14/786	1.92	1.64 (0.97-2.76)	
	Aura (+), Severe	7/492	1.56	0.44 (0.21-0.92)		47/1,700	2.96	1.61 (1.21-2.15)	
DM	Control	8279/211,206	4.50	1 (reference)	.143	4935/125,950	4.4	1 (reference)	.241
	Aura (-), Mild	68/1,615	4.90	0.92 (0.72-1.16)		61/1,856	3.64	0.85 (0.66-1.10)	
	Aura (-), Severe	124/2,109	6.97	1.09 (0.91-1.30)		221/4,467	5.55	1.11 (0.97-1.27)	
	Aura (+), Mild	1/63	1.81	0.29 (0.04-2.02)		1/60	1.86	0.43 (0.06-3.08)	
	Aura (+), Severe	2/108	2.12	0.35 (0.09-1.39)		12/205	6.49	1.35 (0.76-2.37)	
Non-DM	Control	35,319/1,968,003	1.97	1 (reference)		23,729/1,605,314	1.6	1 (reference)	
	Aura (-), Mild	345/14,033	2.67	1.11 (0.99-1.23)		412/23,963	1.84	1.11 (1.00-1.22)	
	Aura (-), Severe	557/14,722	4.18	1.24 (1.14-1.35)		1079/42,264	2.74	1.17 (1.10-1.24)	
	Aura (+), Mild	10/578	1.87	0.82 (0.44-1.53)		18/1,025	1.88	1.31 (0.82-2.08)	
	Aura (+), Severe	15/710	2.30	0.70 (0.42-1.16)		65/2,237	3.12	1.50 (1.18-1.92)	
HTN	Control	24,018/587,607	4.63	1 (reference)	.086	17,936/441,536	4.52	1 (reference)	<.001
	Aura (-), Mild	238/5,155	5.22	1.00 (0.88-1.14)		273/7,673	3.89	0.94 (0.83-1.05)	
	Aura (-), Severe	430/6,908	7.18	1.17 (1.06-1.28)		826/18,188	4.99	1.04 (0.97-1.12)	
	Aura (+), Mild	5/213	2.59	0.52 (0.22-1.24)		10/305	3.62	0.95 (0.51-1.76)	
	Aura (+), Severe	10/337	3.33	0.55 (0.30-1.02)		52/865	6.63	1.45 (1.10-1.9)	
Non-HTN	Control	19,580/1,591,602	1.34	1 (reference)		10,728/1,289,728	0.9	1 (reference)	
	Aura (-), Mild	175/10,493	1.80	1.18 (1.01-1.37)		200/18,146	1.17	1.31 (1.14-1.50)	
	Aura (-), Severe	251/9,923	2.76	1.30 (1.15-1.47)		474/28,543	1.77	1.41 (1.28-1.54)	

	Aura (+), Mild	6/428	1.51	1.00 (0.45-2.22)		9/780	1.23	1.61 (0.84-3.10)	
	Aura (+), Severe	7/481	1.57	0.79 (0.38-1.66)		25/1,577	1.68	1.52 (1.03-2.25)	
Dyslipidemia	Control	10,422/357,027	3.24	1 (reference)	.947	9,711/339,658	3.13	1 (reference)	.509
	Aura (-), Mild	105/3,012	3.85	1.01 (0.83-1.22)		157/5,777	2.93	0.97 (0.83-1.13)	
	Aura (-), Severe	209/3,890	6.05	1.24 (1.08-1.42)		522/13,330	4.27	1.19 (1.09-1.30)	
	Aura (+), Mild	3/148	2.24	0.59 (0.19-1.81)		6/237	2.75	0.97 (0.44-2.17)	
	Aura (+), Severe	5/181	3.11	0.69 (0.29-1.65)		29/647	4.83	1.43 (0.99-2.06)	
Non -DL	Control	33,176/1,822,182	2.0	1 (reference)		18,953/1,391,606	1.48	1 (reference)	
	Aura (-), Mild	308/12,636	2.66	1.09 (0.97-1.22)		316/20,042	1.69	1.12 (1.00-1.25)	
	Aura (-), Severe	472/12,941	4.06	1.20 (1.10-1.31)		778/33,401	2.5	1.13 (1.06-1.22)	
	Aura (+), Mild	8/493	1.75	0.75 (0.38-1.50)		13/848	1.64	1.30 (0.76-2.24)	
	Aura (+), Severe	12/637	2.05	0.60 (0.34-1.06)		48/1,795	2.87	1.50 (1.13-2.00)	
Current smoker	Control	14,735/984,671	1.64	1 (reference)	.987	956/60,031	1.75	1 (reference)	.738
	Aura (-), Mild	115/6,153	2.04	1.04 (0.87-1.25)		16/871	1.99	1.13 (0.69-1.85)	
	Aura (-), Severe	178/5,894	3.34	1.23 (1.06-1.43)		39/1,470	2.88	1.13 (0.82-1.56)	
	Aura (+), Mild	3/237	1.38	0.81 (0.26-2.51)		0/32	0	-	
	Aura (+), Severe	5/283	1.93.	0.66 (0.28-1.59)		4/78	5.69	2.85 (1.07-7.61)	
Non/Ex-smoker	Control	28,863/1,194,538	2.66	1 (reference)		27,708/1,671,233	1.8	1 (reference)	
	Aura (-), Mild	298/9,495	3.44	1.08 (0.96-1.21)		457/24,948	1.96	1.06 (0.97-1.17)	

Aura (-), Severe	503/10,937	5.15	1.20 (1.10-1.32)	1261/45,261	3	1.16 (1.09-1.22)
Aura (+), Mild	8/404	2.14	0.67 (0.33-1.33)	19/1,053	1.94	1.21 (0.77-1.90)
Aura (+), Severe	12/535	2.46	0.61 (0.35-1.08)	73/2,364	3.32	1.44 (1.14-1.81)

Abbreviations: BMI, body mass index; CI, confidence interval; DM, diabetes mellitus; DL, dyslipidemia; HR, hazard ratio; HTN, hypertension; IR, incidence rate; p-y, person-year.

*Multivariate Cox regression model for adjustment included covariates as follows: age, smoking habit, alcohol consumption level, regular physical activity, low household income level, body mass index (BMI), DM, hypertension, dyslipidemia, and estimated glomerular filtration rate.

II. Supplementary Figures

Figure S1. Cumulative Incidence of Atrial Fibrillation in Total Migraine Patients

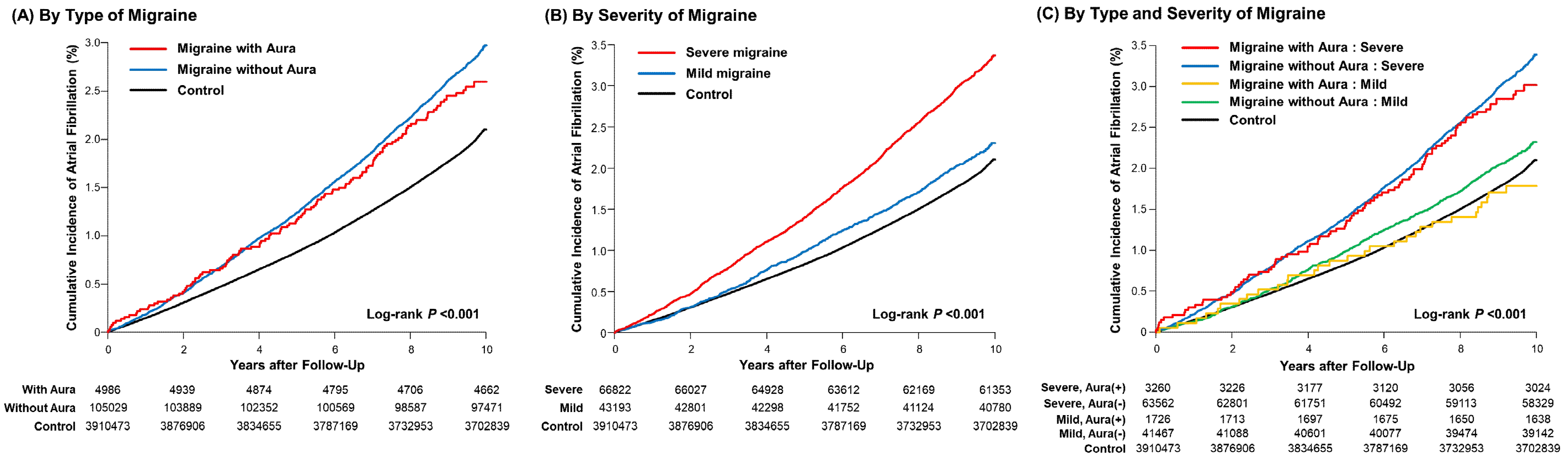

Cumulative hazard curves for AF according to the (A) type, (B) severity, and (C) combination of type and severity of migraine in total population are shown.

Abbreviations: AF, atrial fibrillation

Figure S2. Sex-Stratified Cumulative Incidence of Atrial Fibrillation According to Type and Severity of Migraine

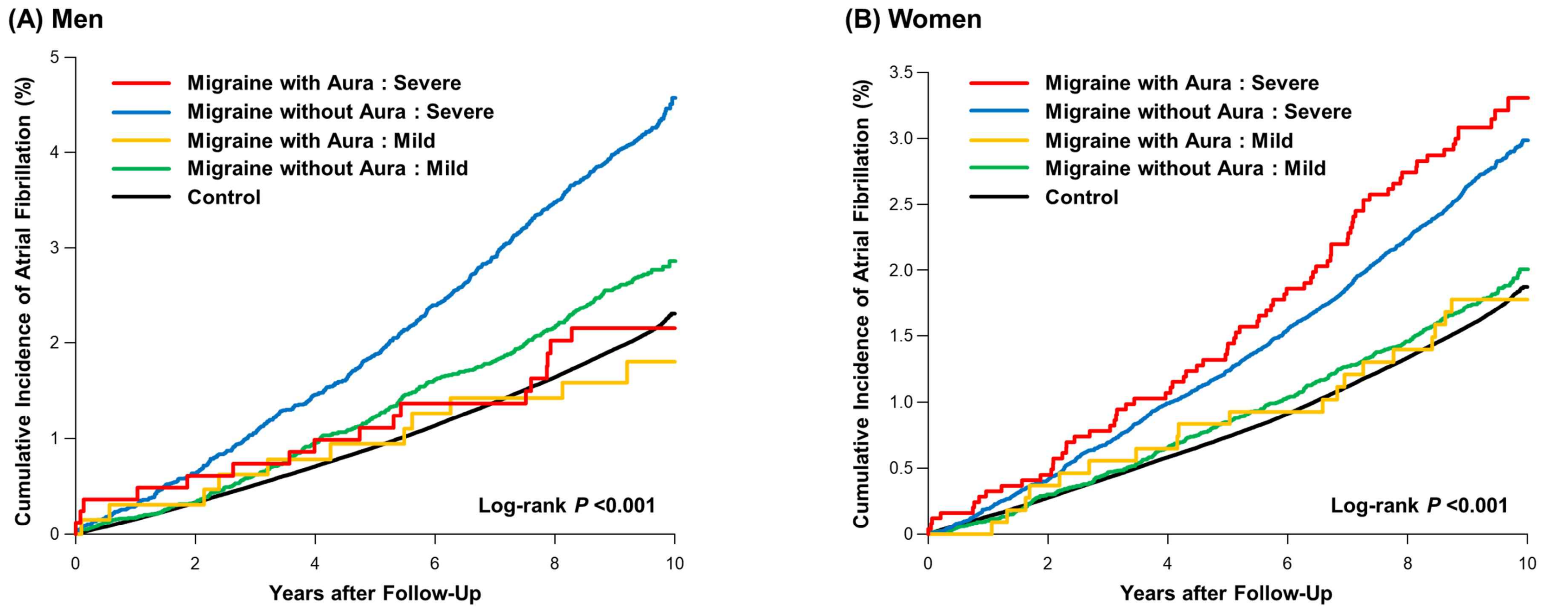

(A) Men

	0	2	4	6	8	10
**Severe, Aura(+)**	818	806	792	768	749	737
**Severe, Aura(-)**	16831	16493	16054	15524	14999	14715
**Mild, Aura(+)**	641	637	628	615	606	603
**Mild, Aura(-)**	15648	15443	15167	14876	14593	14430
**Control**	2179209	2156901	2129029	2098232	2064046	2045088

(B) Women

	0	2	4	6	8	10
**Severe, Aura(+)**	2442	2420	2385	2352	2307	2287
**Severe, Aura(-)**	46731	46308	45697	44968	44114	43614
**Mild, Aura(+)**	1085	1076	1069	1060	1044	1035
**Mild, Aura(-)**	25819	25645	25434	25201	24881	24712
**Control**	1731264	1720005	1705626	1688937	1668907	1657751

Cumulative hazard curves for AF according to the combination of type and severity of migraine in (A) men and (B) women are shown.

Figure S3. Sex-Stratified Cumulative Incidence of Atrial Fibrillation According to Severity of Migraine

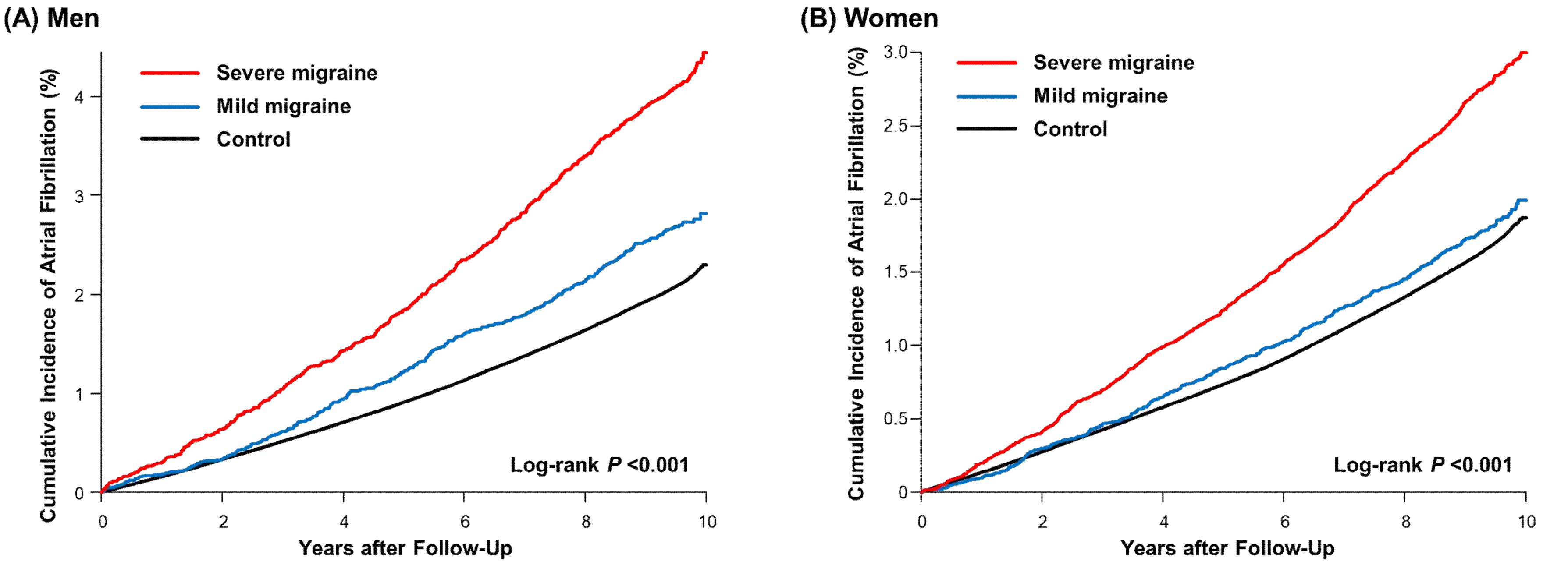

(A) Men

	0	2	4	6	8	10
**Severe**	17649	17299	16846	16292	15748	15452
**MIld**	16289	16080	15795	15491	15199	15033
**Control**	2179209	2156901	2129029	2098232	2064046	2045088

(B) Women

	0	2	4	6	8	10
**Severe**	49173	48728	48082	47320	46421	45901
**Mild**	26904	26721	26503	26261	25925	25747
**Control**	1731264	1720005	1705626	1688937	1668907	1657751

Cumulative hazard curves for AF according to the severity of migraine in (A) men and (B) women are shown.

Abbreviations are as in Figure S1.
